# Squamous Cell Carcinoma in Never Smokers: An Insight into SMARCB1 Loss

**DOI:** 10.3390/ijms25158165

**Published:** 2024-07-26

**Authors:** Akshay J. Patel, Hanan Hemead, Hannah Jesani, Andrea Bille, Philippe Taniere, Gary Middleton

**Affiliations:** 1 Institute of Immunology and Immunotherapy, College of Medical and Dental Sciences, University of Birmingham, Vincent Drive, Edgbaston, Birmingham B15 2TT, UK; g.middleton@bham.ac.uk; 2 Department of Thoracic Surgery, University Hospitals Birmingham, Birmingham B15 2GW, UK; hanan.hemead@nhs.net (H.H.); hannah.jesani@nhs.net (H.J.); 3 Department of Thoracic Surgery, Guy’s Hospital, Guy’s and St. Thomas’ NHS Foundation Trust, London SE1 9RT, UK; andrea.bille@nhs.net; 4 Department of Cellular Histopathology, University Hospitals Birmingham, Birmingham B15 2GW, UK; philippe.taniere@uhb.nhs.uk

**Keywords:** squamous cell carcinoma (SqCC), non-small cell lung cancer (NSCLC), lung cancer in never smokers (LCINS), SMARCB1, SWF/INI complex, BAF complex

## Abstract

Lung cancer remains the leading cause of cancer-related mortality worldwide, with non-small cell lung cancer (NSCLC) constituting 85% of cases. Among NSCLCs, squamous cell carcinoma (SqCC) is strongly associated with smoking. However, lung cancer in never smokers (LCINS) represents approximately 25% of lung cancer cases globally and shows increasing incidence, particularly in East Asia. LCINS-SqCC is less well-characterized, especially regarding its genomic alterations and their impact on clinical outcomes. We conducted a retrospective analysis over a 20-year period (July 2003–July 2023) at two major tertiary centers in the UK. The cohort included 59 patients with LCINS-SqCC who underwent radical surgical resection. Data collected included demographic information, comorbidities, histopathological details, and outcome metrics such as disease-free and overall survival. Molecular sequencing of tumor specimens was performed to identify genomic aberrations. The cohort had a median age of 71 years (IQR 62–77) and a median BMI of 25.4 (IQR 22.8–27.8), with a slight male predominance (53%). The majority of patients (93%) had a preoperative MRC of 1–2. Recurrent disease was observed in 23 patients (39%), and 32 patients (54%) had died at a median follow-up of 3 years. Median disease-free survival was 545 days (IQR 132–1496), and overall survival was 888 days (IQR 443–2071). Preoperative creatinine levels were higher in patients who experienced recurrence (*p* = 0.037). Molecular analysis identified biallelic SMARCB1 loss in two younger patients, associated with rapid disease progression despite R0 resection. These patients’ tumors were PDL1-negative, TTF-1-negative, and positive for cytokeratin, CD56, and p40. SMARCB1-deficient SqCC in never smokers represents a highly aggressive variant with poor disease-free survival, highlighting the importance of integrating advanced molecular diagnostics in clinical practice. This study underscores the necessity for personalized treatment strategies, including targeted therapies such as EZH2 inhibitors and immune checkpoint blockade, to address the unique molecular pathways in SMARCB1-deficient cancers. Further clinical trials are essential to optimize therapeutic approaches for this challenging subgroup of lung cancer.

## 1. Introduction

Lung cancer remains the leading cause of cancer-related mortality worldwide, accounting for greater than 1 million deaths [1]. Non-small cell lung cancer (NSCLC) accounts for 85% of all lung cancers and comprises two major subtypes; adenocarcinoma and squamous cell carcinoma (SqCC) [2]. Tobacco smoke is considered the single greatest risk factor in the etiopathogenesis of lung cancers, and this association is much stronger in the SqCC subtype [3]. Lung cancer in never smokers (LCINS), however, still accounts for roughly 25% of all lung cancer worldwide [4], and epidemiological data [5] have shown an increasing trend in the incidence of LCINS over the last two decades. There is a geographic disparity in that the prevalence of LCINS is much higher in the east Asian subcontinent [6]. 

LCINS is a heterogeneous entity with several risk factors having been identified in the etiopathogenesis; namely high-risk occupations, low BMI, indoor biomass use without proper ventilation, air pollution (PM_2.5_), and positive family history with germline mutations [7,8]. The majority of these data emerge from the adenocarcinoma subtype, which is the predominant histology seen in never smokers. Biologically, these adenocarcinomas differ greatly from adenocarcinomas seen in ever smokers, with differences in the proportion of oncogenic driver mutations such as EGFR, KRAS, and BRAF [9,10,11]; these seem to predominate in never smokers, females, and those of east Asian descent [10,12]. A lot of these oncogenic driver mutations are clinically actionable, and analysis of RNA-sequencing data has shown these never-smoker adenocarcinomas to possess distinct immune transcriptional subtypes that vary in their expression of clinically relevant immune checkpoint molecules and immune cell composition [13].

These mutations have also been described in SqCC, however to a much lesser extent, particularly in the western world. There is a paucity of data closely examining the clinicopathological and genetic features of this subgroup [14]. LCINS do possess distinct genomic alterations that impact survival [15] and data from Huang et al. [14] have shown that SqCC in never smokers is more poorly differentiated with a higher number of genomic aberrations that are potentially targetable. With the advent of lung cancer screening [16,17], there is likely to be an increased detection of lung cancers in never smokers, and understanding the clinical relevance of genomic alterations in less understood subtypes such as SqCC is likely to be hugely impactful. 

In this study, we aim to explore the natural history of our cohort of SqCC in never smokers and understand the clinical relevance of any specific genomic aberrations. 

## 2. Results

Between July 2003 and July 2023, we identified 59 patients with no significant smoking history who had undergone radical lung resection for SqCC. The median age of the cohort was 71 (IQR 62–77), with a median BMI of 25.4 (IQR 22.8–27.8). There was a slight male preponderance (53%, n = 31). The cohort was quite functionally robust, with 93% of cohort having a preoperative MRC of 1–2 (n = 55). The presence of COPD, ischemic heart disease, and diabetes was found in 11, 5, and 9 patients, respectively. At median follow-up of 3 years, 23 patients (39%) had developed recurrent disease, and 32 patients (54%) had died. Median disease-free and overall survival were 545 (132–1496) and 888 (443–2071) days, respectively. 

We compared baseline characteristics between those who recurred and those who did not and found no significant differences in age, sex, BMI or co-morbidities. Pre-operative creatinine was significantly higher in those who recurred (80 versus 60, *p* = 0.037). There were no differences in any pre-operative or histopathological criteria between those who died and those who are alive at long-term follow-up. 

We undertook molecular sequencing analysis of the tumor cells on the post-operative specimen blocks to ascertain if any driver mutations could be found particularly in those patients who recurred early post-operatively and indeed those who were diagnosed at a young age. In two patients, aged 32 and 39 at the time of resection, we found biallelic loss of SMARCB1. Both these patients were previously fit and well, presented with incidental findings of limited stage I node negative lung cancer, and, following resection, succumbed to relapse within 56 and 148 days, respectively, despite R0 resections. Both cancers were PDL1 < 0%, without any other actionable driver mutations, cytokeratin, CD56- and p40-positive, and TTF-1 negative. 

**Figure 1:**
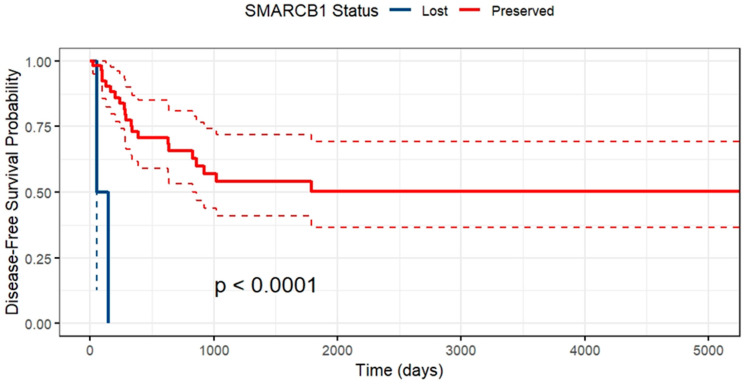
Kaplan–Meier curve demonstrating disease-free survival between SMARCB1 loss and preservation.

SMARCB1 loss had no impact on overall survival, which is unsurprising given the small sample size, but significantly impacted disease-free survival (HR 16.67 95% CI 3.03–100; *p* = 0.01). Figure 1 illustrates the difference in disease-free survival between preservation and loss of SMARCB1 with significantly reduced disease-free survival with loss of function of this gene by log-rank testing.

Table 1 below highlights the clinical pathway for the two young patients who were found to have SMARCB1-deficient carcinomas. Despite the highlighted stark differences in the staging and presentation of these two cases, the commonality of the SMARCB1 loss resulted in high propensity for aggression and early recurrence.

## 3. Discussion

Lung cancer accounts for the highest number of cancer-related deaths worldwide [1]. For both males and females, breast, lung, and prostate cancers account for the most diagnosed malignancies globally. According to 2022 census data [1], progress has stagnated for breast and prostate cancers but strengthened for lung cancer, coinciding with changes in medical practice related to cancer screening and/or treatment. LCINS is fast becoming more prevalent globally. Data from murine models and resected human samples have shown that particulate matter less than or equal to 2.5 μM (PM_2.5_) is associated with lung cancer risk. EGFR-driven cancers in never smokers showed a significant association of PM_2.5_ and the incidence of lung cancer in multiple cohorts. This was reinforced by functional murine data where exposure to air pollutants resulted in macrophage infiltration and subsequent interleukin 1β release, which fueled tumorigenesis [8]. Whilst the majority of LCINS cases tend to be on the adenocarcinoma spectrum, SqCC is still seen in never smokers. Oncogenic driver mutations such as those seen in exons 19 and 21 of the EGFR gene have been reported in SqCC [18], but this is quite infrequent. The morphologic features that suggest squamous differentiation include intercellular bridging, squamous pearl formation, and individual cell keratinization. These can be quite apparent in well-differentiated tumors; however, in poorly differentiated tumors, they are difficult to find [19]. SqCC can have numerous subtypes, but these do not properly address the morphologic spectrum let alone the genomic profile of these tumors; hence, current standards do not allow for meaningful correlations with clinical, prognostic, or molecular features.

Loss of SMARCB1 has been identified as the sole mutation in a number of rare adult and pediatric cancers. These tend to be highly aggressive, being refractory to most treatments including surgery, chemotherapy, and radiotherapy, and hence confer a very poor prognosis [20]. The protein SMARCB1 (SNF5/INI/BAF47/SWI/SNF-Related, Matrix-Associated, Actin-Dependent Regulator of Chromatin, Subfamily B, Member 1) is a highly conserved core subunit of the mammalian ATP-dependent BAF chromatin remodeling complex, which is a key regulator of nucleosome repositioning and gene expression [20]. SMARCB1-deficient cancers are characterized by biallelic loss and inactivation of this tumor suppressor gene; this is particularly noted in rare and aggressive childhood cancers such as rhabdoid tumor of the kidney, atypical teratoid rhabdoid tumors, synovial sarcoma, and myoepithelial carcinomas of the lung [21,22,23,24,25,26].

Biological relevance of this tumor suppressor gene was first highlighted in a murine model where loss of SMARCB1 was shown to result in a highly penetrant cancer predisposition with 100% of mice developing mature CD8+ T cell lymphoma or rare rhabdoid tumors with a median onset of 11 weeks [27]. Loss of SMARCB1 causes the widespread loss of BAF localization, which leads to unchecked PRC2-mediated transcriptional repression at enhancers and promoters [28]. On a molecular level, SMARCB1 has been shown to regulate the critical tumor suppressor, p16 (also known as p16^INK4a^); p16 is a cyclin-dependent kinase inhibitor that binds to CDK4/6 and prevents activation of the CDK4/6-cyclin D1 complex [29]. SMARCB1-deficient cells have reduced p16 expression, which ultimately leads to increased cellular proliferation due to unchecked S phase progression [20]. Additionally, *Myc*, which is considered a transcriptional activator that potentiates oncogenic transformation when over-expressed, is thought to be regulated by SMARCB1 such that its activation targets are suitably inhibited to prevent neoplastic change [30]. 

SMARCB1 loss has been described in lung cancer as isolated case reports. SMARCA4 loss has been noted in conjunction with an EGFR mutation [26]. SMARCB1 has been noted in STK11 deficient de-differentiated lung cancer [25]. In the setting of thoracic neoplasms, it has been described that SMARCA4 loss correlates with poorer outcome and expression of SOX2, CD34, and SALL4, and SMARCB1 loss is mainly seen in thoracic neoplasms of a mesenchymal lineage [31]. Both cases described from our series are virtually identical to that described by Rickard et al. [24] in that the majority of cells were p40-positive and uniformly positive for cytokeratins. The patient described in this report [24] was initially treated with Paclitaxel, Carboplatin, and Ipilimumab and Nivolumab and responded to the first piece of this, but then progressed on the immunotherapy alone, very much implying chemotherapy responsiveness. They were then treated with Gemcitabine/Carboplatin and sustained a good metabolic response in both the primary and metastatic site, and at the time of the report were still responding. Further to this, a partial response to combination neoadjuvant Pembrolizumab and combination platinum doublet therapy has been reported in a case of advanced SMARCB1-deficient cancer [25]. 

There are now significant advances in the therapeutic targeting of SMARCB1-deficient cancers, gained through understanding of the molecular biology of this protein. EZH2 inhibition in particular has been biologically and clinically viable, having shown efficacy in SMARCB1-deficient sarcomas [32]. The FDA-approved drug Tazemetostat is a potent EZH2 inhibitor [33] and was employed in these sarcoma patients [32]. 

The BAF complex opposes the repressive PRC2 complex to facilitate chromatin decompaction, nucleosome remodeling by promoting sliding, or ejection of nucleosomes; this facilitates coordination of gene expression. PRC2 complexes induce transcriptional repression by catalyzing methylation of histone H3 on lysine 27 [20,23]. The BAF and PRC2 complex essentially exist in a state of functional antagonism. Upon loss of SMARCB1, there is no ability to oppose PRC2-induced repression of the nucleosome, hence no ability to express BAF-target genes. EZH2 is the catalytic subunit of PRC2, which can be targeted by FDA-approved agents such as Tazemetostat [33,34]. 

Other targets that have been proposed include dual blockade of tyrosine kinase receptors; PDGF-Rα/β and FGFR-2, which have been shown to be co-activated in rhabdoid tumors and can be targeted by the tyrosine kinase inhibitor (TKI), Ponatinib [35]. GBAF, another member of the ATP-dependent chromatin remodeling family and an identified subcomplex of BAF, has been proposed as another target in atypical teratoid rhabdoid tumors (ATRT) for anti-tumor potential [36]. In vitro work has shown that proliferative growth in ATRT and synovial sarcomas is dependent on residual GBAF functional activity and hence targeting selective subunits of the GBAF subcomplex; namely, BRD9 or GLTSCR1 can reduce cellular viability in these solid tumors [20,36]. MYC inhibition has been shown to reduce ATRT tumor growth in vivo and employ the BET inhibitor; IQ1 in orthotopic ATRT xenografts has mimicked the effect of direct MYC inhibition [37]. 

Immune checkpoint blockade is another area that is being trialed in SMARCB1-deficient cancers. Although the two patients in our series were PDL1-negative, data from a pediatric series [38] showed PDL1 positivity in 47% of tumor samples with concurrent SMARCB1 loss. In SMARCB1-deficient sarcomas, several clinical trials have reported variable success rates with checkpoint blockade as monotherapy or as combination therapy [39]. Some studies (NCT03277924 and NCT02332668) have shown best response durations of up to 17 months [40,41]. In the setting of unrestricted EZH2 activity, there is likely to be repression of class I and class II and indeed PDL1 expression, hence reducing the efficacy of checkpoint blockade. However, a large proportion of SMARCB1-deficient tumors do demonstrate a degree of immune infiltration [39]. Clinical trials have been conducted to address the biological plausibility of concurrent EZH2 inhibition with Tazemetostat and immune checkpoint blockade [23]. A phase I/II study to assess the value of combining immune checkpoint inhibitors (nivolumab and ipilimumab) with Tazemetostat has been launched (NCT05407441) [42] for the treatment of a variety of SMARCB1/INI1- or SMARCA4-deficient tumors [23]. EZH2 inhibition has been shown to increase the infiltration of CD8+ T cells in ovarian cancer models [43], and comparisons of tumor biopsy samples obtained prior to and during Tazemetostat revealed a substantial increase in intra-tumoral and stromal infiltrates of CD8+ cytotoxic and FOXP3+ regulatory T cells, together with an enhanced expression of the PD-1 and LAG3 immune-checkpoint proteins on T cells [39]. Phase II trial data (CAIRE trial; NCT04705818) [44] evaluating the association of Tazemetostat and anti-PD-L1 (Durvalumab) in solid tumors will hopefully elucidate these biological relationships further. 

The most commonly accepted mechanistic insight into SMARCB1-deficient cancers is that the loss of the SMARC protein results in the pathological manifestation of cancer. Recent data have shown that the gene DDB1-CUL4-associated factor 5 (DCAF5) is required for the survival of SMARCB1-mutant cancers and actually promotes the degradation of incompletely assembled SWI/SNF complexes in the absence of SMARCB1. After depletion of DCAF5, SMARCB1-deficient SWI/SNF complexes reaccumulate, bind to target loci, and restore SWI/SNF-mediated gene expression to levels that are sufficient to reverse the cancer state, including in vivo [45]. Consequently, cancer results not from the loss of SMARCB1 function, but rather from DCAF5-mediated degradation of SWI/SNF complexes. These data indicate that therapeutic targeting of ubiquitin-mediated quality-control factors may effectively reverse the malignant state of some cancers driven by disruption of tumor suppressor complexes [45].

## 4. Methods and Materials

We performed a retrospective analysis over a 20-year period (July 2003–July 2023) from two major tertiary centers in the United Kingdom. We interrogated our data series to identify all the LCINS-SqCC patients who had undergone radical surgical resection.

Inclusion Criteria:

1. Any patient who had undergone radical surgical resection for squamous cell carcinoma of the lung in the time period.
2. Never smoking status.

Exclusion Criteria:

1. Non-squamous histology.

The time period was dictated by the availability and completeness of data from the electronic patients’ records (EPR) and the national audit database. Data were reported in accordance with the STROBE guidelines [46,47]. Our primary aim was to identify any key histopathological features that were unique to this cohort and if said features were linked to survival. We collected data on demographic features (age, sex, BMI), comorbidities, histopathological data (IASLC staging post-operatively), and outcome data (disease-free and overall survival and length of hospital stay). Smoking status was described and modeled according to the criteria set out in previous epidemiological studies to ensure we were truly capturing never smokers [48].

### Statistical Analysis

For continuous variables, results are expressed as means and standard deviations or medians and IQR depending on the distribution of the data. For categorical variables, we reported counts and percentages. For continuous data, group comparison was carried out using a Student’s *t*-test or Mann–Whitney test depending on the distribution of data. Normality was assessed using Shapiro–Wilk testing. Group differences for categorical data were assessed using the chi squared test of independence or Fisher’s exact test for low frequencies. Time-to-event data were analyzed using Cox proportional hazards modeling. Survival analyses were carried out using *survminer* and displayed using the *ggsurv/ggplot2* packages [49,50,51]. Data were displayed using the *gtsummary* package [52]. The tests were considered significant at *p* < 0.05. All analyses were performed using R programming software (v4.0.3) in R studio.

## 5. Conclusions

This study elucidates the aggressive nature of SMARCB1-deficient squamous cell carcinoma (SqCC) in never smokers, highlighting a significant impact on disease-free survival and underscoring the urgent need for advanced molecular diagnostics and targeted therapies. The identification of biallelic SMARCB1 loss in younger patients with LCINS-SqCC, associated with rapid relapse despite standard resection, illustrates a critical prognostic biomarker. It is important, however, to recognize that this analysis is limited by the small series and retrospective nature. 

As lung cancer in never smokers, particularly SqCC, continues to rise globally, the integration of molecular profiling into routine clinical practice is imperative for the development of personalized treatment strategies and improved patient outcomes. Advanced therapeutic approaches, including EZH2 inhibition and immune checkpoint blockade, show promise in addressing the distinct molecular pathways disrupted in SMARCB1-deficient cancers, paving the way for future clinical trials to optimize treatment efficacy in this challenging subset of lung cancer.

## Figures and Tables

**Figure 2:**
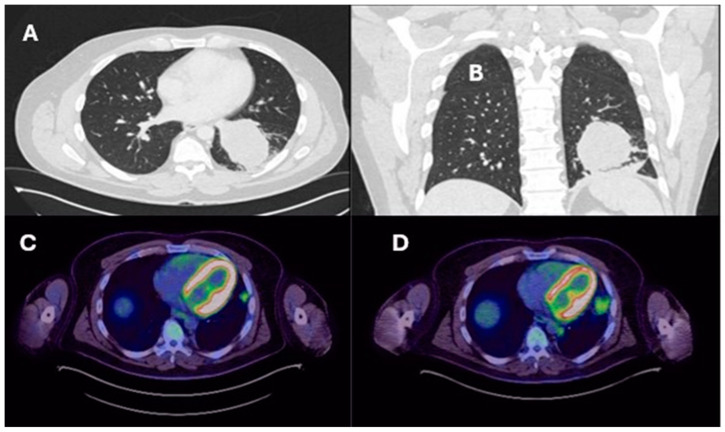
Contrast-enhanced chest CT, axial section demonstrating a large left lower lobe lung cancer (**A**), coronal view (**B**), post-resectional recurrence on PET-CT, axial section (**C**), and recurrence in the station 8 paraoesophageal region (**D**).

**Figure 3:**
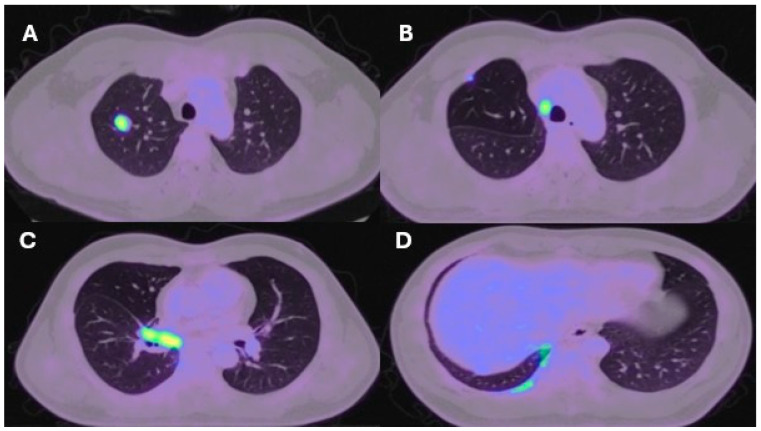
PET-CT axial slice of a right upper lobe stage I lung cancer (**A**). Post-operative recurrence in the upper paratracheal regions on PET-CT (**B**). Recurrence at the right hilum (**C**). Recurrence in the posterior ribs on the right-hand side (**D**).

**Table 1:** Schema of clinical pathway for two SMARCB1 loss patients.

Patient 1	Patient 2
36-year-old male. Never smoker. BMI 31.1. No significant co-morbidities. No history of cancer.	39-year-old male. Never smoker. BMI 27.4. No significant co-morbidities. No history of cancer.
Presented with dull left sided chest pain in September 2022.	Incidental finding of lung mass in right upper lobe after investigation for abdominal pain.
Stage 2b NSCLC.	Stage 1B NSCLC.
Lung resection November 2022—Left lower lobectomy + lymph node dissection: pT3pN0 R0 resection. SMARCB1-deficient, PDL1-negative, TTF-1 negative, CD56-positive, cytokeratin positive. Pre-operative CT axial and coronal slices are shown in Figure 2A and Figure 2B, respectively.	Lung resection January 2022—right upper lobectomy + lymph node dissection: pT1cpN0 R0 resection. SMARCB1-deficient, PDL1 negative, TTF-1 negative, CD56-positive, cytokeratin-positive. Pre-operative axial PET slice shown in Figure 3A.
Histopathological Features: 58 mm size tumor, lymphovascular and perineural invasion seen, no spread through airways (STAS), no breach of visceral pleura.	Histopathological Features: 21 mm size tumor, no lymphovascular and perineural invasion seen, no spread through airways (STAS), no breach of visceral pleura.
Recurrence December 2022—started 4 cycles of Gemcitabine/Cisplatin. Post-operative recurrence demonstrated in PET slices (Figure 2C,D)	Recurrence June 2022—progressive disease noted on CTPA (performed for SOB, pyrexia) at the right hilum, with soft tissue thickening at resection margins. Commenced on Pembrolizumab/Paclitaxel/Cisplatin with systemic intent. Recurrence shown in station 4R (Figure 3B), at the right hilum (Figure 3C) and in the right posterior bony skeleton in ribs 6–9 (Figure 3D).
Progressive disease in pleura and mediastinum April 2023—compassionate release form to administer Tazemetostat.	Chemotherapy stopped due to severe allergic reaction to drugs—November 2022. Resolution of soft tissue thickening on PET but with new pleural metastases and bulky paraoesophageal lymphadenopathy. Further maintenance Pembrolizumab only.
Increased burden of disease in left hemithorax August 2023—compassionate release of combination checkpoint blockade: Nivolumab/Ipilimumab, Tazemetostat stopped.	Slight progression on CT January 2023. Patient opted for surgical resection of metastatic sites abroad.
November 2023—three cycles of checkpoint blockade but developed severe rib pain due to a medial 4th destroying lesion (pure progressive disease), given 20 Gy in 5# of palliative radiotherapy.	Further lung resection March 2023—multiple wedge resections of RUL and RML. Radical lymphadenectomy and resection of tumor deposits in the chest wall, pericardium, and mediastinum and sheath of the SVC.
December 2023—all treatment stopped in light of progression and episode of viral pneumonia. Referred to hospice for palliation.	Patient currently under strict surveillance and has relocated abroad.
February 2024—Under palliative care team with good symptom control.	

## Data Availability

All data and materials are available upon reasonable request from the corresponding author.
